# Supplementary figures and images for: The combination of quercetin and leucine synergistically improves grip strength by attenuating muscle atrophy by multiple mechanisms in mice exposed to cisplatin
Source: PLoS One. 2023 Sep 12;18(9):e0291462. doi: 10.1371/journal.pone.0291462 (PMC10497166; doi:10.1371/journal.pone.0291462)

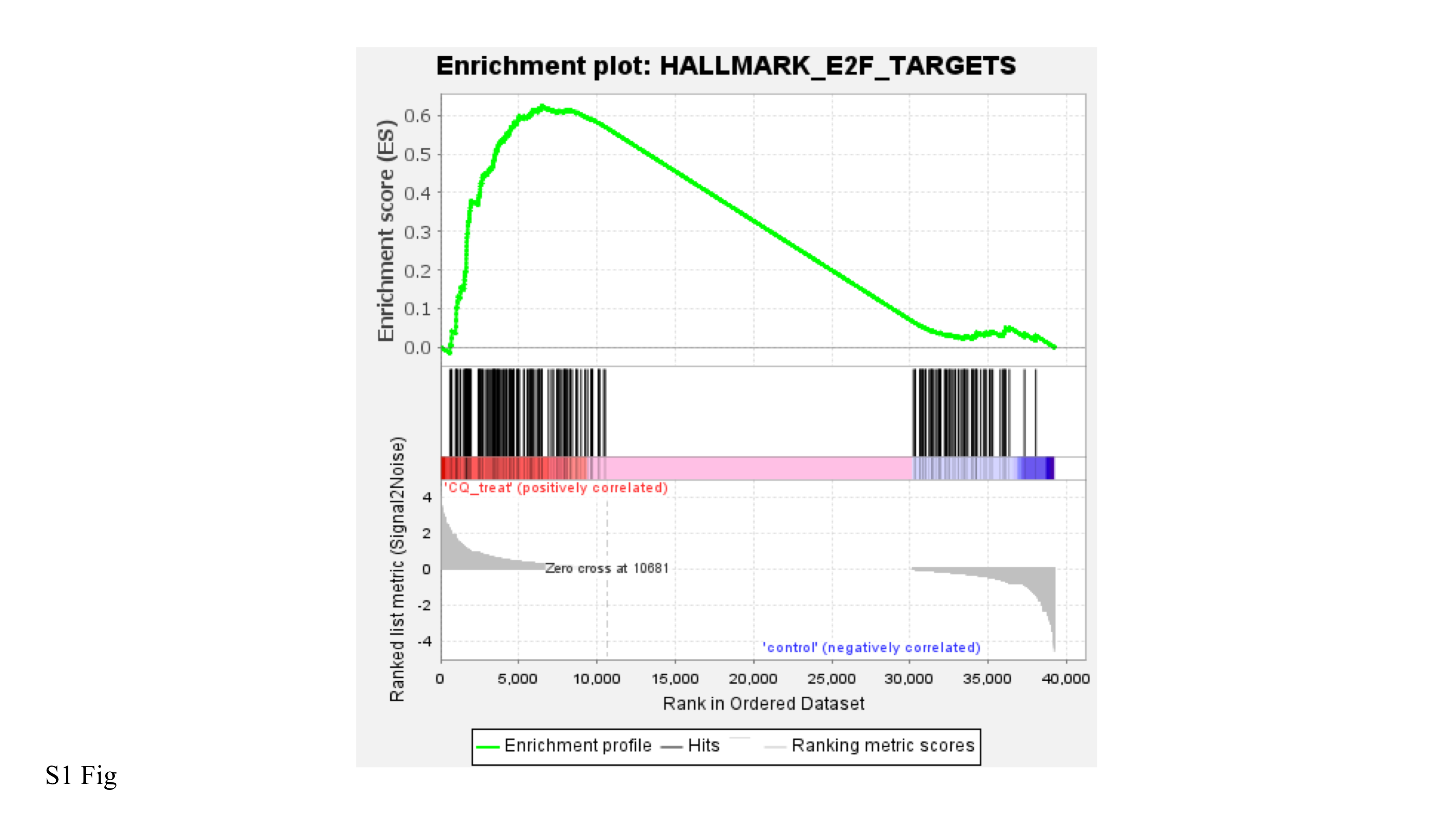

Supplement: S1 Fig — The cells were incubated in Eagle’s Minimum Essential Medium supplemented with 10% (v/v) fetal bovine serum and 1% penicillin-streptomycin at 37 ˚C in a humidified atmosphere of 5% CO2. After co-incubation with cisplatin (1 μM) and quercetin (5 μM) for 48 hours, the total RNA of cells was collected for the next generation sequencing (NGS) and GSEA. (TIFF) [file pone.0291462.s001.tiff]

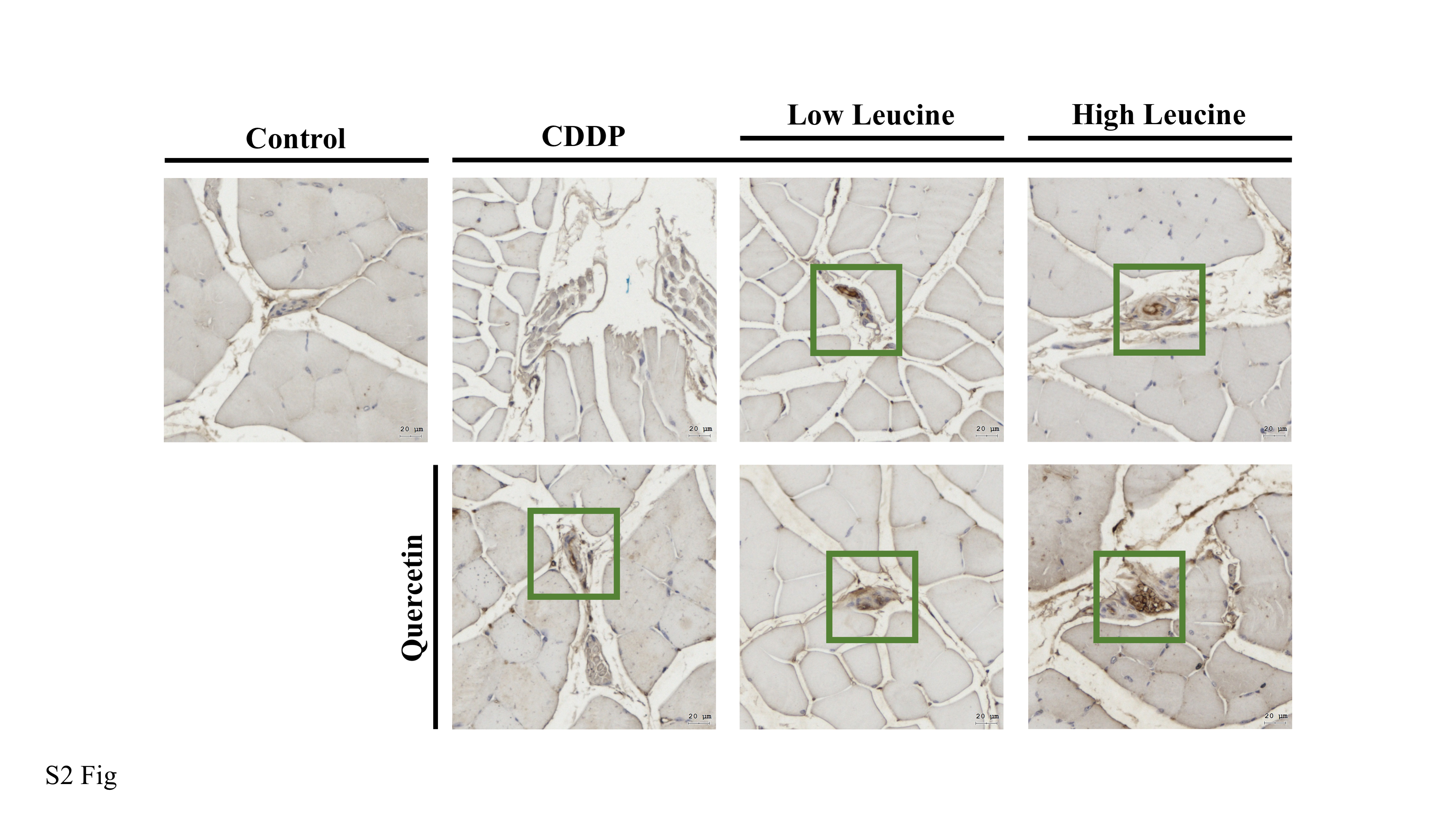

Supplement: S2 Fig — Immunohistochemical staining was performed by the UltraView Universal DAB Detection Kit (Roche, Switzerland) and Ki-67 antibody (cat #: 12202, Cell Signaling Technology) and the sections were examined using the Tissue Cytometer (TissueGnostics, Vienna, Australia; magnification, x200). The nuclei were stained in blue with Hematoxylin and Ki-67-positive cells were stained brown. Bar in the picture is 20 μm and the area framed by the rectangles represents Ki-67-positive cells. (TIFF) [file pone.0291462.s002.tiff]

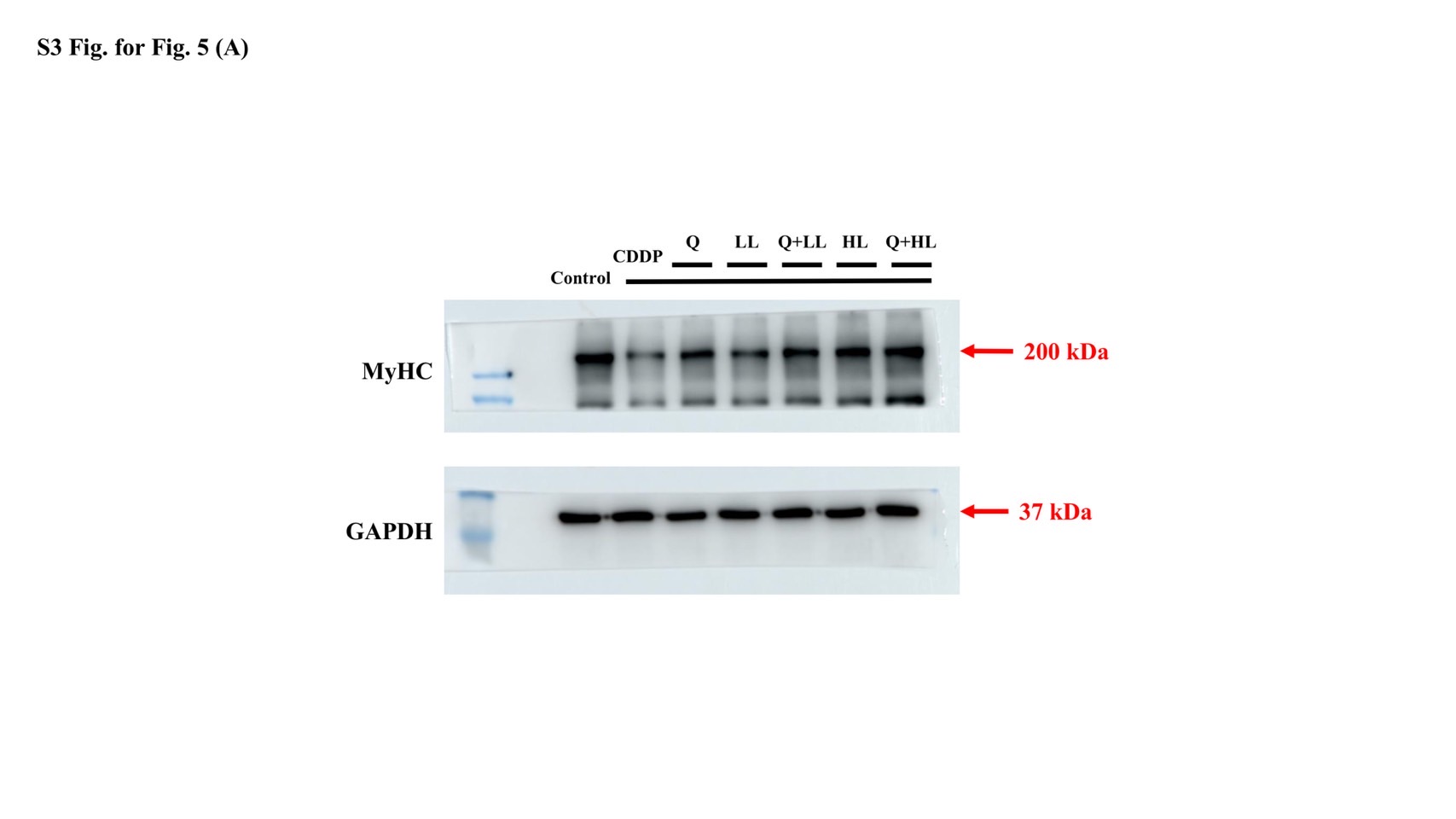

Supplement: S3 Fig — (JPG) [file pone.0291462.s003.jpg]

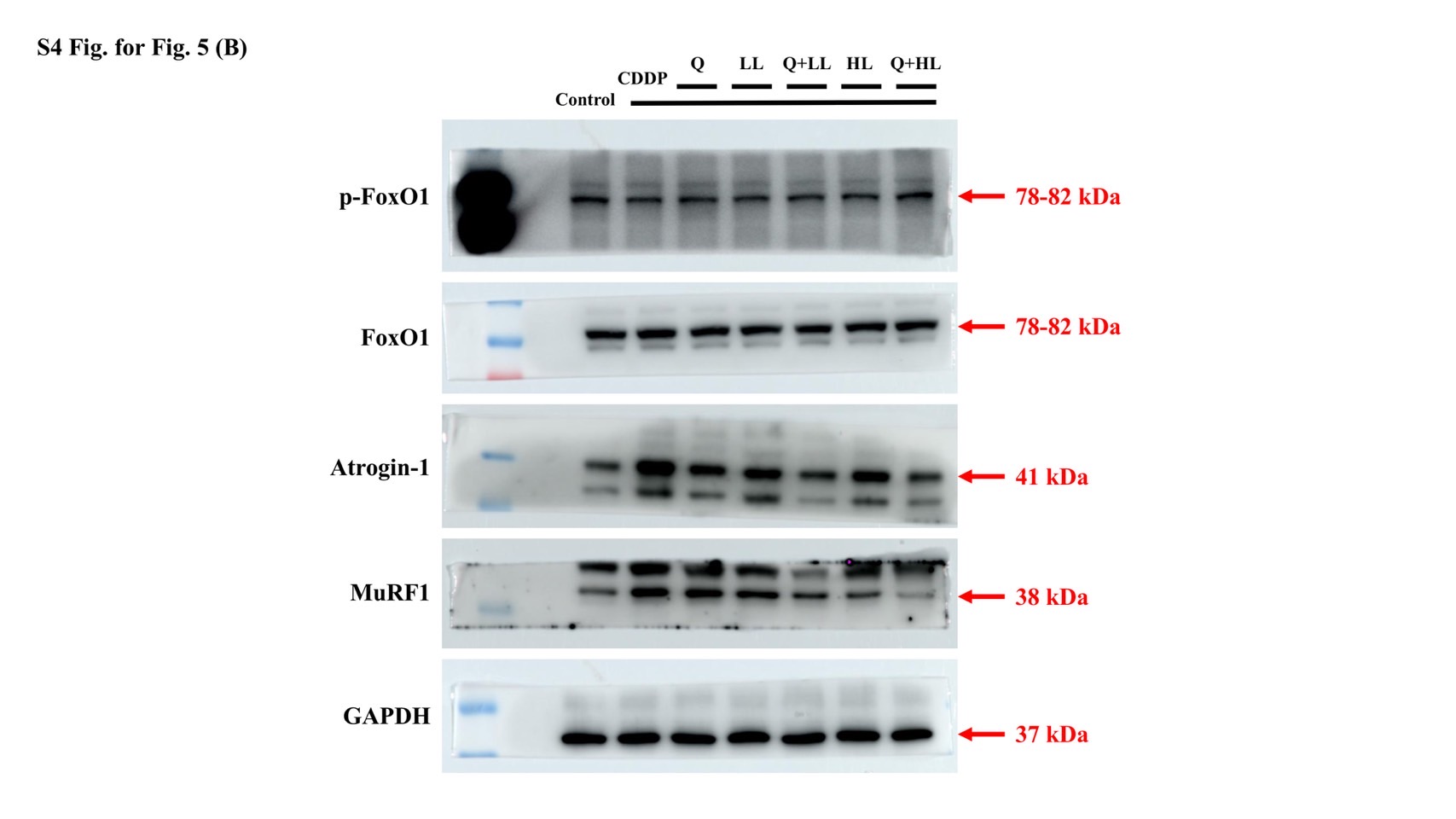

Supplement: S4 Fig — (JPG) [file pone.0291462.s004.jpg]

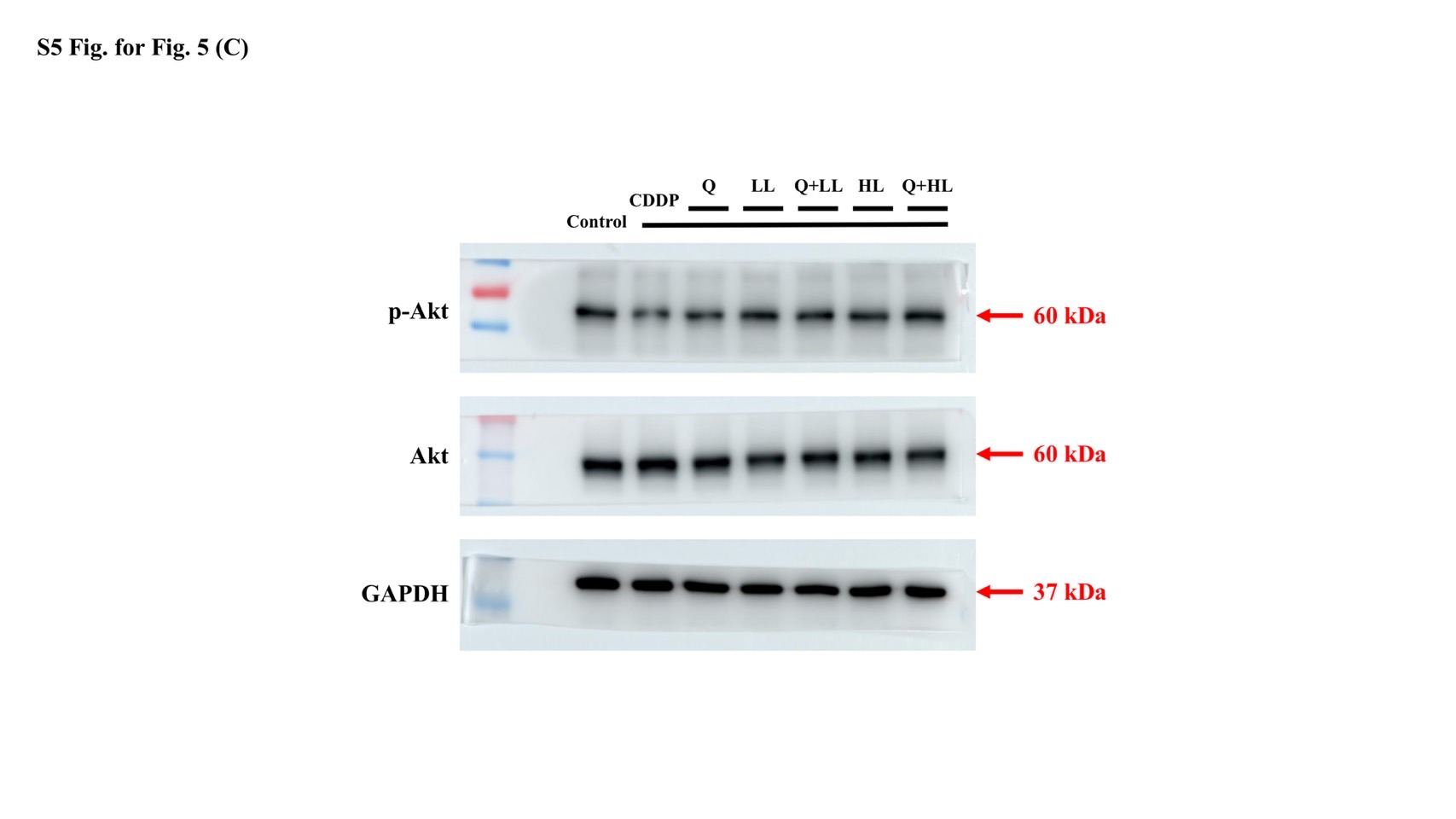

Supplement: S5 Fig — (JPG) [file pone.0291462.s005.jpg]

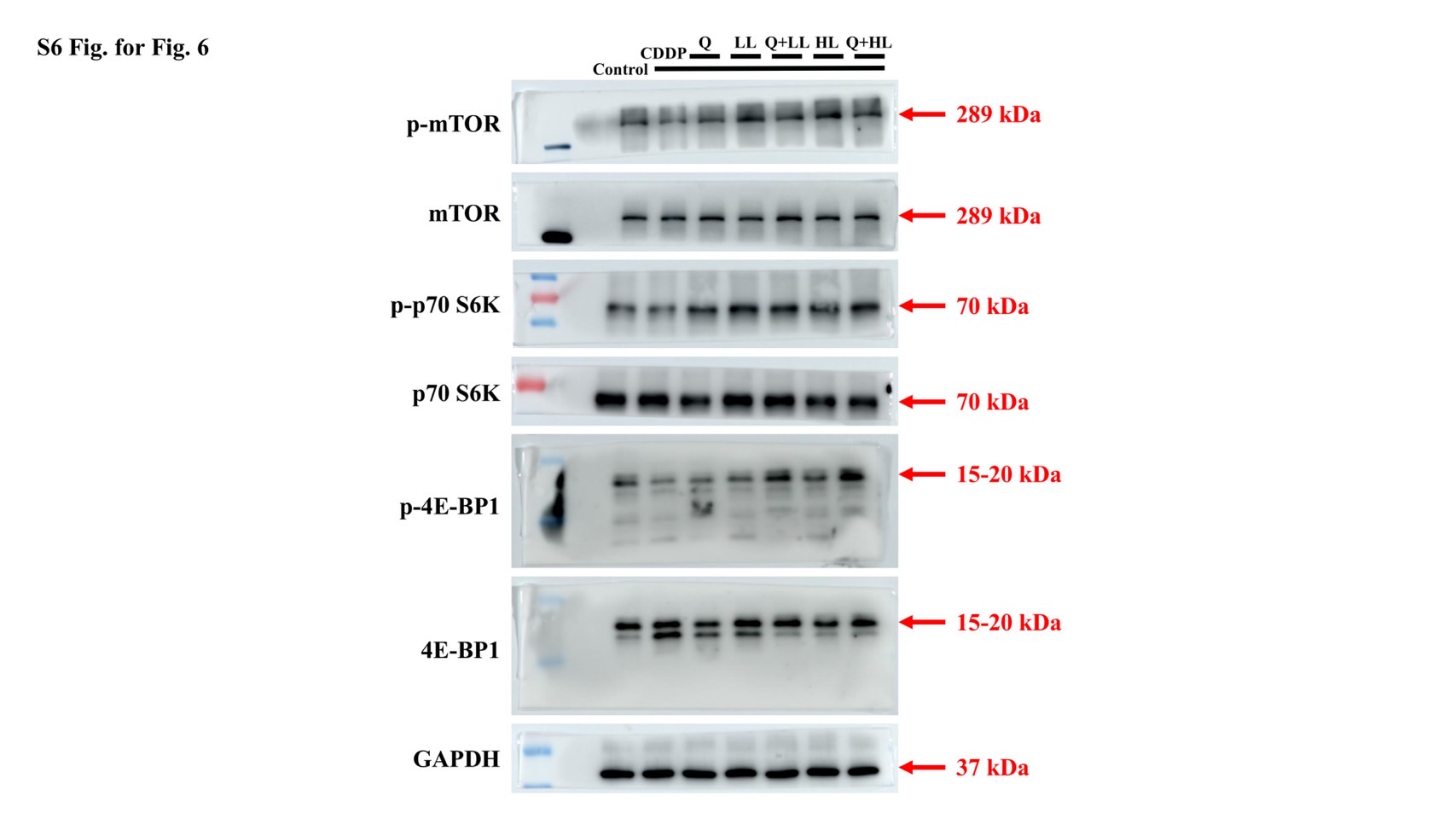

Supplement: S6 Fig — (JPG) [file pone.0291462.s006.jpg]

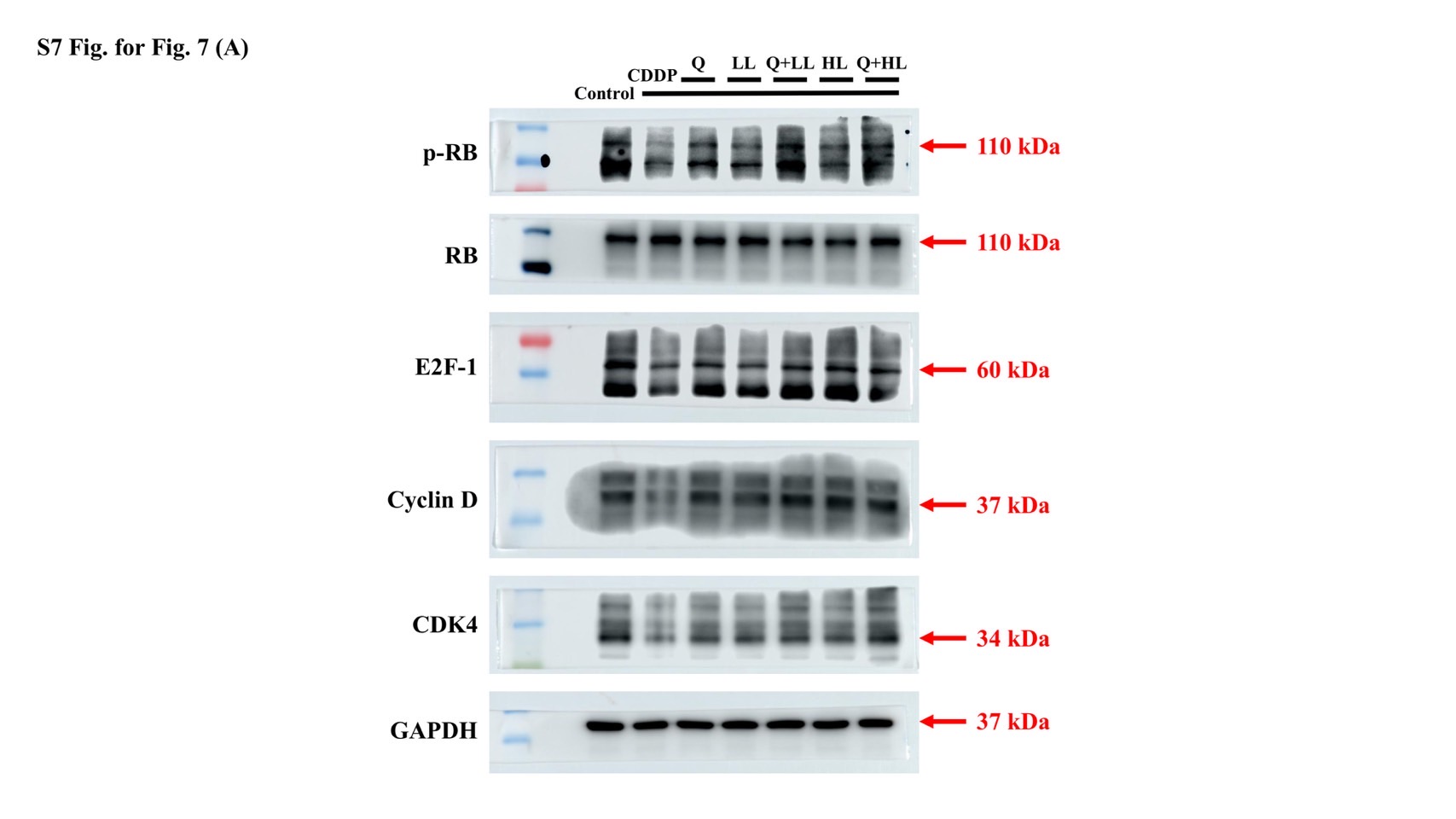

Supplement: S7 Fig — (JPG) [file pone.0291462.s007.jpg]

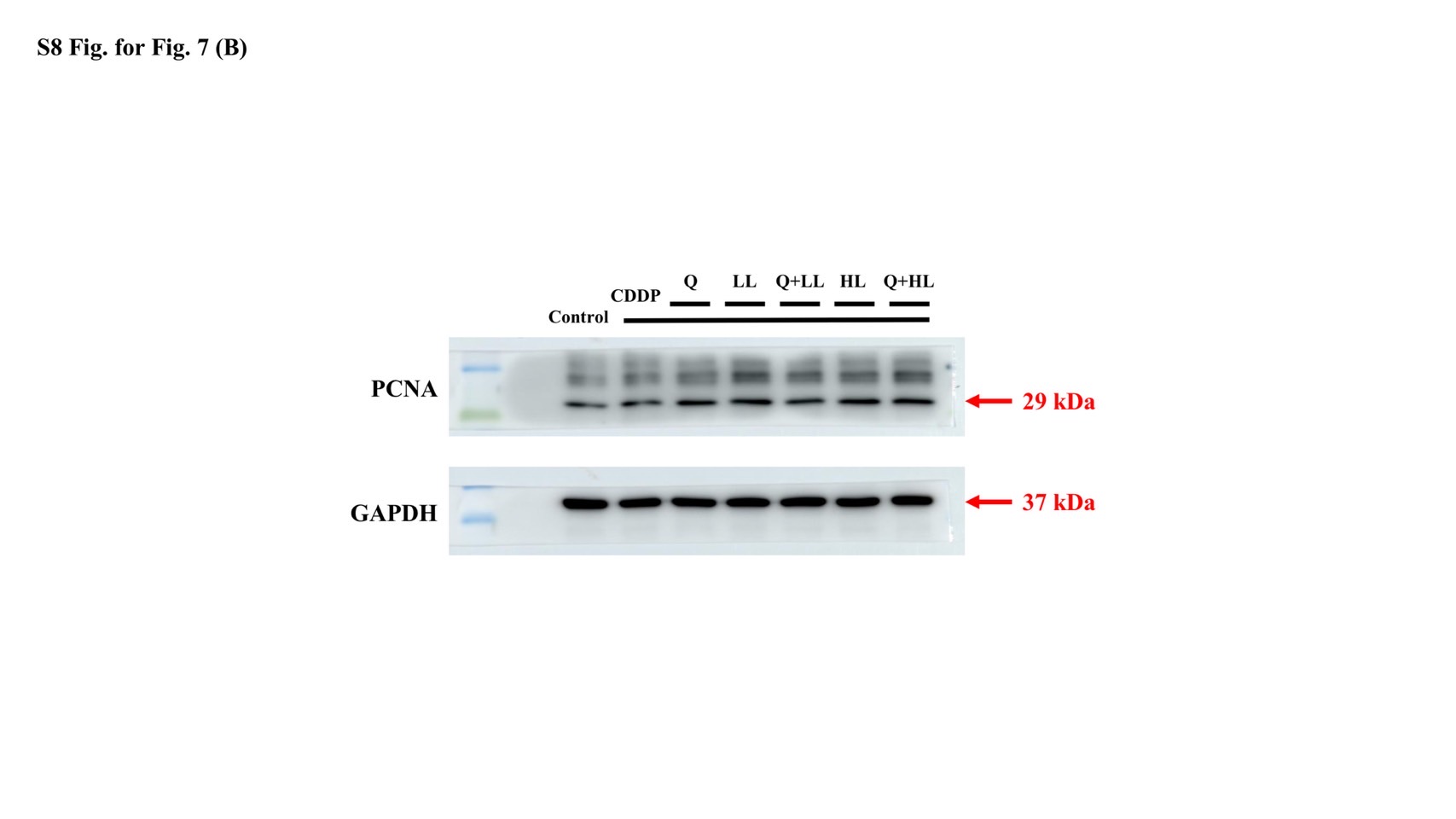

Supplement: S8 Fig — (JPG) [file pone.0291462.s008.jpg]
