# Supplementary material for: The combination of quercetin and leucine synergistically improves grip strength by attenuating muscle atrophy by multiple mechanisms in mice exposed to cisplatin
Source: PLoS One. 2023 Sep 12;18(9):e0291462. doi: 10.1371/journal.pone.0291462 (PMC10497166; doi:10.1371/journal.pone.0291462)
Supplement: S1 File — Comparison between observed inhibition and expected inhibition of quercetin (Q) in combination with low dose (LL) or high dose of leucine (HL) on the levels of parameters determined in BALB/c mice (S1-S9 Tables) or tumor-bearing nude mice (S10 Table) exposed to cisplatin. (DOCX) [file pone.0291462.s009.docx]

S1 Table. Comparison between observed inhibition and expected inhibition of quercetin (Q) in combination with low dose (LL) or high dose of leucine (HL) on body weight and food intake in BALB/c mice exposed to cisplatin (CDDP).

| Treatment | Observed inhibition (%)^a^ | Expected inhibition (%)^a^ | *P* value^b^ |
| --- | --- | --- | --- |
| Body weight | | | |
| CDDP | - | - | - |
| + Q | 12.2 ± 6.4 | - | - |
| + LL | 15.6 ± 6.3 | - | - |
| + HL | 17.6 ± 8.8 | - | - |
| + Q + LL | 26.0 ± 6.8 | 27.8 | 0.592 |
| + Q + HL | 33.3 ± 10.6 | 29.9 | 0.509 |
| Food intake | | | |
| CDDP | - | - | - |
| + Q | 7.4 ± 2.0 | - | - |
| + LL | 32.3 ± 3.6 | - | - |
| + HL | 29.2 ± 3.3 | - | - |
| + Q + LL | 46.2 ± 7.5 | 39.8 | 0.274 |
| + Q + HL | 46.5 ± 9.6 | 36.6 | 0.215 |

^a^The observed inhibition (%) of one parameter was calculated as ∣(the value of treatment- the value of CDDP)∣/the value of CDDP × 100%; the expected inhibition (%) was calculated as the observed inhibition of quercetin + the observed inhibition of leucine (LL/HL).

^b^Statistical analysis was performed by the one-sample t-test.

S2 Table. Comparison between observed inhibition and expected inhibition of quercetin (Q) in combination with low dose (LL) or high dose of leucine (HL) on maximum grip strength and locomotor activity in BALB/c mice exposed to cisplatin (CDDP).

| Treatment | Observed inhibition (%) ^a^ | Expected inhibition (%)^a^ | *P* value^b^ |
| --- | --- | --- | --- |
| Maximum grip strength | | | |
| CDDP | - | - | - |
| + Q | 7.0 ± 4.6 | - | - |
| + LL | 11.2 ± 6.7 | - | - |
| + HL | 15.1 ± 3.1 | - | - |
| + Q + LL | 26.6 ± 7.2 | 18.2 | 0.035* |
| + Q + HL | 37.2 ± 7.8 | 22.1 | 0.005* |
| Locomotor activity | | | |
| CDDP | - | - | - |
| + Q | 209.3 ± 59.5 | - | - |
| + LL | 411.6 ± 51.2 | - | - |
| + HL | 439.5 ± 77.3 | - | - |
| + Q + LL | 525.6 ± 83.4 | 620.9 | 0.038 |
| + Q + HL | 551.2 ± 44.7 | 648.8 | 0.003 |

^a^The observed inhibition (%) of one parameter was calculated as ∣(the value of treatment- the value of CDDP)∣/the value of CDDP × 100%; the expected inhibition (%) was calculated as the observed inhibition of quercetin + the observed inhibition of leucine (LL/HL).

^b^Statistical analysis was performed by the one-sample t-test.

S3 Table. Comparison between observed inhibition and expected inhibition of quercetin (Q) in combination with low dose (LL) or high dose of leucine (HL) on epididymal fat and total muscle weight in BALB/c mice exposed to cisplatin (CDDP).

| Treatment | Observed inhibition (%) ^a^ | Expected inhibition (%)^a^ | *P* value^b^ |
| --- | --- | --- | --- |
| Epididymal fat weight | | | |
| CDDP | - | - | - |
| + Q | 122.9 ± 44.7 | - | - |
| + LL | 226.9 ± 68.4 | - | - |
| + HL | 287.3 ± 120.4 | - | - |
| + Q + LL | 298.2 ± 44.3 | 349.8 | 0.06 |
| + Q + HL | 422.2 ± 149.6 | 410.2 | 0.886 |
| Total muscle weight | | | |
| CDDP | - | - | - |
| + Q | 8.2 ± 7.6 | - | - |
| + LL | 11.4 ± 3.6 | - | - |
| + HL | 10.5 ± 5.7 | - | - |
| + Q + LL | 19.1 ± 8.1 | 19.7 | 0.894 |
| + Q + HL | 20.9 ± 7.9 | 18.7 | 0.57 |

^a^The observed inhibition (%) of one parameter was calculated as ∣(the value of treatment- the value of CDDP)∣/the value of CDDP × 100%; the expected inhibition (%) was calculated as the observed inhibition of quercetin + the observed inhibition of leucine (LL/HL).

^b^Statistical analysis was performed by the one-sample t-test.

S4 Table. Comparison between observed inhibition and expected inhibition of quercetin (Q) in combination with low dose (LL) or high dose of leucine (HL) on triceps, quadriceps, gastrocnemius, soleus, and tibialis anterior muscle weight in BALB/c mice exposed to cisplatin (CDDP).

| Treatment | Observed inhibition (%)^a^ | Expected inhibition (%)^a^ | *P* value^b^ |
| --- | --- | --- | --- |
| Triceps muscle weight | | | |
| CDDP | - | - | - |
| + Q | 8.4 ± 9.3 | - | - |
| + LL | 11.6 ± 1.2 | - | - |
| + HL | 9.2 ± 8.5 | - | - |
| + Q + LL | 15.0 ± 8.8 | 20.0 | 0.27 |
| + Q + HL | 19.6 ± 9.8 | 17.5 | 0.654 |
| Quadriceps muscle weight | | | |
| CDDP | - | - | - |
| + Q | 13.0 ± 7.9 | - | - |
| + LL | 15.9 ± 4.4 | - | - |
| + HL | 14.3 ± 7.4 | - | - |
| + Q + LL | 24.3 ± 10.6 | 28.9 | 0.389 |
| + Q + HL | 29.1 ± 8.0 | 27.3 | 0.647 |
| Gastrocnemius muscle weight | | | |
| CDDP | - | - | - |
| + Q | 9.0 ± 7.9 | - | - |
| + LL | 12.4 ± 5.3 | - | - |
| + HL | 13.6 ± 4.1 | - | - |
| + Q + LL | 19.5 ± 8.6 | 21.4 | 0.646 |
| + Q + HL | 21.4 ± 6.3 | 22.5 | 0.703 |
| Soleus muscle weight | | | |
| CDDP | - | - | - |
| + Q | 7.7 ± 8.0 | - | - |
| + LL | 9.6 ± 11.0 | - | - |
| + HL | 17.3 ± 4.3 | - | - |
| + Q + LL | 9.6 ± 11.0 | 17.3 | 0.192 |
| + Q + HL | 32.7 ± 8.0 | 25.0 | 0.099 |
| Tibialis anterior muscle weight | | | |
| CDDP | - | - | - |
| + Q | 6.3 ± 4.9 | - | - |
| + LL | 7.3 ± 6.6 | - | - |
| + HL | 7.9 ± 3.0 | - | - |
| + Q + LL | 12.3 ± 13.0 | 13.6 | 0.83 |
| + Q + HL | 12.3 ± 6.3 | 14.2 | 0.523 |

^a^The observed inhibition (%) of one parameter was calculated as ∣(the value of treatment- the value of CDDP)∣/the value of CDDP × 100%; the expected inhibition (%) was calculated as the observed inhibition of quercetin + the observed inhibition of leucine (LL/HL).

(Continued)

^b^Statistical analysis was performed by the one-sample t-test.

S5 Table. Comparison between observed inhibition and expected inhibition of quercetin (Q) in combination with low dose (LL) or high dose of leucine (HL) on muscle fiber size in quadriceps muscle in BALB/c mice exposed to cisplatin (CDDP).

| Treatment | Observed inhibition (%) ^a^ | Expected inhibition (%) ^a^ | *P* value^b^ |
| --- | --- | --- | --- |
| Muscle fiber size | | | |
| CDDP | - | - | - |
| + Q | 29.3 ± 7.8 | - | - |
| + LL | 30.5 ± 5.3 | - | - |
| + HL | 31.9 ± 6.0 | - | - |
| + Q + LL | 63.0 ± 9.2 | 59.9 | 0.438 |
| + Q + HL | 80.2 ± 14.6 | 61.2 | 0.024* |

^a^The observed inhibition (%) of one parameter was calculated as ∣(the value of treatment- the value of CDDP)∣/the value of CDDP × 100%; the expected inhibition (%) was calculated as the observed inhibition of quercetin + the observed inhibition of leucine (LL/HL).

^b^Statistical analysis was performed by the one-sample t-test.

* The combined effect was synergistic.

S6 Table. Comparison between observed inhibition and expected inhibition of quercetin (Q) in combination with low dose (LL) or high dose of leucine (HL) on relative protein expression of MyHC, p-FoxO1/FoxO1, atrogin-1, MuRF1 and p-Akt/Akt in gastrocnemius muscle in BALB/c mice exposed to cisplatin (CDDP).

| Treatment | Observed inhibition (%) ^a^ | Expected inhibition (%) ^a^ | *P* value^b^ |
| --- | --- | --- | --- |
| MyHC | | | |
| CDDP | - | - | - |
| + Q | 23.1 ± 9.4 | - | - |
| + LL | 13.2 ± 7.6 | - | - |
| + HL | 25.3 ± 8.4 | - | - |
| + Q + LL | 42.0 ± 16.1 | 36.3 | 0.533 |
| + Q + HL | 55.5 ± 3.8 | 48.4 | 0.035* |
| p-FoxO1/FoxO1 | | | |
| CDDP | - | - | - |
| + Q | 7.7 ± 1.7 | - | - |
| + LL | 6.4 ± 3.4 | - | - |
| + HL | 18.8 ± 5.8 | - | - |
| + Q + LL | 18.2 ± 5.2 | 14.1 | 0.214 |
| + Q + HL | 22.8 ± 5.0 | 26.6 | 0.235 |
| Atrogin-1 | | | |
| CDDP | - | - | - |
| + Q | 21.6 ± 7.5 | - | - |
| + LL | 18.2 ± 12.6 | - | - |
| + HL | 20.5 ± 10.0 | - | - |
| + Q + LL | 37.0 ± 6.9 | 39.8 | 0.489 |
| + Q + HL | 39.2 ± 4.4 | 42.1 | 0.282 |
| MuRF1 | | | |
| CDDP | - | - | - |
| + Q | 18.7 ± 4.9 | - | - |
| + LL | 17.1 ± 3.8 | - | - |
| + HL | 24.2 ± 5.3 | - | - |
| + Q + LL | 24.9 ± 3.9 | 35.8 | 0.012 |
| + Q + HL | 32.6 ± 2.7 | 43.0 | 0.004 |
| p-Akt/Akt | | | |
| CDDP | - | - | - |
| + Q | 22.8 ± 6.4 | - | - |
| + LL | 42.9 ± 6.4 | - | - |
| + HL | 36.0 ± 4.5 | - | - |
| + Q + LL | 43.3 ± 12.5 | 65.7 | 0.038 |
| + Q + HL | 48.2 ± 8.8 | 58.8 | 0.095 |

^a^The observed inhibition (%) of one parameter was calculated as ∣(the value of treatment- the value of CDDP)∣/the value of CDDP × 100%; the expected inhibition (%) was calculated as the observed inhibition of quercetin + the observed inhibition of leucine (LL/HL).

(Continued)

^b^Statistical analysis was performed by the one-sample t-test.

* The combined effect was synergistic.

S7 Table. Comparison between observed inhibition and expected inhibition of quercetin (Q) in combination with low dose (LL) or high dose of leucine (HL) on relative protein expression of p-mTOR/mTOR, p-p70 S6K/p70 S6K, and p-4E-BP1/4E-BP1 in gastrocnemius muscle in BALB/c mice exposed to cisplatin (CDDP).

| Treatment | Observed inhibition (%)^a^ | Expected inhibition (%)^a^ | *P* value^b^ |
| --- | --- | --- | --- |
| p-mTOR/mTOR | | | |
| CDDP | - | - | - |
| + Q | 12.8 ± 5.7 | - | - |
| + LL | 37.7 ± 22.1 | - | - |
| + HL | 40.4 ± 17.5 | - | - |
| + Q + LL | 44.3 ± 14.7 | 50.5 | 0.462 |
| + Q + HL | 41.9 ± 17.8 | 53.2 | 0.292 |
| p-p70 S6K/p70 S6K | | | |
| CDDP | - | - | - |
| + Q | 34.0 ± 5.5 | - | - |
| + LL | 61.9 ± 11.4 | - | - |
| + HL | 68.4 ± 5.1 | - | - |
| + Q + LL | 61.8 ± 11.7 | 95.9 | 0.01 |
| + Q + HL | 69.7 ± 9.0 | 102.4 | 0.005 |
| p-4E-BP1/4E-BP1 | | | |
| CDDP | - | - | - |
| + Q | 57.3 ± 7.4 | - | - |
| + LL | 34.3 ± 9.9 | - | - |
| + HL | 65.0 ± 9.7 | - | - |
| + Q + LL | 78.9 ± 4.6 | 91.6 | 0.011 |
| + Q + HL | 98.4 ± 14.8 | 122.3 | 0.048 |

^a^The observed inhibition (%) of one parameter was calculated as ∣(the value of treatment- the value of CDDP)∣/the value of CDDP × 100%; the expected inhibition (%) was calculated as the observed inhibition of quercetin + the observed inhibition of leucine (LL/HL).

^b^Statistical analysis was performed by the one-sample t-test.

S8 Table. Comparison between observed inhibition and expected inhibition of quercetin (Q) in combination with low dose (LL) or high dose of leucine (HL) on relative protein expression of p-RB/RB, E2F-1, cyclin D, CDK4, and PCNA in gastrocnemius muscle in BALB/c mice exposed to cisplatin (CDDP).

| Treatment | Observed inhibition (%)^a^ | Expected inhibition (%)^a^ | *P* value^b^ |
| --- | --- | --- | --- |
| p-RB/RB | | | |
| CDDP | - | - | - |
| + Q | 39.1 ± 10.4 | - | - |
| + LL | 42.9 ± 6.3 | - | - |
| + HL | 43.7 ± 11.6 | - | - |
| + Q + LL | 42.8 ± 7.2 | 82.0 | 0.002 |
| + Q + HL | 43.0 ± 6.0 | 82.8 | 0.001 |
| E2F-1 | | | |
| CDDP | - | - | - |
| + Q | 10.9 ± 12.6 | - | - |
| + LL | 1.8 ± 15.6 | - | - |
| + HL | 25.3 ± 7.9 | - | - |
| + Q + LL | 25.2 ± 8.7 | 12.7 | 0.065 |
| + Q + HL | 26.7 ± 18.7 | 36.2 | 0.385 |
| Cyclin D | | | |
| CDDP | - | - | - |
| + Q | 14.7 ± 9.7 | - | - |
| + LL | 20.9 ± 8.8 | - | - |
| + HL | 23.3 ± 8.5 | - | - |
| + Q + LL | 27.6 ± 8.2 | 35.6 | 0.145 |
| + Q + HL | 30.5 ± 13.6 | 38.0 | 0.349 |
| CDK4 | | | |
| CDDP | - | - | - |
| + Q | 25.8 ± 7.6 | - | - |
| + LL | 24.1 ± 9.0 | - | - |
| + HL | 25.3 ± 10.2 | - | - |
| + Q + LL | 45.4 ± 15.9 | 49.9 | 0.612 |
| + Q + HL | 44.3 ± 8.0 | 51.1 | 0.188 |
| PCNA | | | |
| CDDP | - | - | - |
| + Q | 28.0 ± 21.1 | - | - |
| + LL | 41.4 ± 20.0 | - | - |
| + HL | 42.1 ± 8.6 | - | - |
| + Q + LL | 46.7 ± 17.7 | 69.3 | 0.083 |
| + Q + HL | 42.7 ± 15.4 | 70.1 | 0.037 |

^a^The observed inhibition (%) of one parameter was calculated as ∣(the value of treatment- the value of CDDP)∣/the value of CDDP × 100%; the expected inhibition (%) was calculated as the observed inhibition of quercetin + the observed inhibition of leucine (LL/HL).

(Continued)

^b^Statistical analysis was performed by the one-sample t-test.

S9 Table. Comparison between observed inhibition and expected inhibition of quercetin (Q) in combination with low dose (LL) or high dose of leucine (HL) on the levels of glycogen, MCP-1, TNF-α, IL-6, and IL-1β in triceps muscle in BALB/c mice exposed to cisplatin (CDDP).

| Treatment | Observed inhibition (%) ^a^ | Expected inhibition (%) ^a^ | *P* value ^b^ |
| --- | --- | --- | --- |
| Glycogen | | | |
| CDDP | - | - | - |
| + Q | 22.0 ± 12.4 | - | - |
| + LL | 26.4 ± 14.4 | - | - |
| + HL | 36.8 ± 8.0 | - | - |
| + Q + LL | 28.9 ± 20.0 | 48.4 | 0.146 |
| + Q + HL | 73.2 ± 20.8 | 58.8 | 0.26 |
| MCP-1 | | | |
| CDDP | - | - | - |
| + Q | 20.4 ± 2.8 | - | - |
| + LL | 11.7 ± 4.3 | - | - |
| + HL | 15.0 ± 3.2 | - | - |
| + Q + LL | 29.9 ± 3.2 | 32.1 | 0.364 |
| + Q + HL | 34.5 ± 1.8 | 35.4 | 0.485 |
| TNF-α | | | |
| CDDP | - | - | - |
| + Q | 41.4 ± 2.9 | - | - |
| + LL | 31.4 ± 3.7 | - | - |
| + HL | 44.1 ± 4.6 | - | - |
| + Q + LL | 52.5 ± 3.4 | 72.8 | 0.009 |
| + Q + HL | 59.0 ± 3.7 | 85.5 | 0.006 |
| IL-6 | | | |
| CDDP | - | - | - |
| + Q | 25.6 ± 3.9 | - | - |
| + LL | 25.0 ± 3.6 | - | - |
| + HL | 28.8 ± 1.5 | - | - |
| + Q + LL | 37.6 ± 0.6 | 50.6 | 0.001 |
| + Q + HL | 43.7 ± 2.3 | 54.4 | 0.015 |
| IL-1β | | | |
| CDDP | - | - | - |
| + Q | 26.3 ± 4.9 | - | - |
| + LL | 25.8 ± 6.7 | - | - |
| + HL | 31.6 ± 1.5 | - | - |
| + Q + LL | 44.9 ± 3.9 | 52.0 | 0.086 |
| + Q + HL | 47.8 ± 6.1 | 57.8 | 0.104 |

^a^The observed inhibition (%) of one parameter was calculated as ∣(the value of treatment- the value of CDDP)∣/the value of CDDP × 100%; the expected inhibition (%) was calculated as the observed inhibition of quercetin + the observed inhibition of leucine (LL/HL).

(Continued)

^b^Statistical analysis was performed by the one-sample t-test.

S10 Table. Comparison between observed inhibition and expected inhibition of quercetin (Q) in combination with a high dose of leucine (HL) on food intake, tumor size, as well as body, epididymal fat, and gastrocnemius muscle weight in tumor-bearing nude mice exposed to cisplatin (CDDP).

| Treatment | Observed inhibition (%)^a^ | Expected inhibition (%)^a^ | *P* value^b^ |
| --- | --- | --- | --- |
| Food intake | | | |
| CDDP | - | - | - |
| + Q | 0.6 ± 20.5 | - | - |
| + HL | 0.9 ± 19.7 | - | - |
| + Q + HL | 11.0 ± 22.5 | 1.5 | 0.351 |
| Tumor size^c^ | | | |
| CDDP | - | - | - |
| + Q | -22.8 ± 26.5 | - | - |
| + HL | 2.4 ± 31.6 | - | - |
| + Q + HL | -20.8 ± 22.6 | -20.4 | 0.966 |
| Body weight | | | |
| CDDP | - | - | - |
| + Q | 8.6 ± 6.9 | - | - |
| + HL | 12.6 ± 2.9 | - | - |
| + Q + HL | 13.8 ± 10.1 | 21.2 | 0.332 |
| Epididymal fat weight | | | |
| CDDP | - | - | - |
| + Q | 171.9 ± 48.1 | - | - |
| + HL | 156.9 ± 57.8 | - | - |
| + Q + HL | 335.3 ± 99.2 | 328.8 | 0.919 |
| Gastrocnemius muscle weight | | | |
| CDDP | - | - | - |
| + Q | 7.4 ± 9.9 | - | - |
| + HL | 8.4 ± 3.7 | - | - |
| + Q + HL | 24.9 ± 14.1 | 15.8 | 0.38 |

^a^The observed inhibition (%) of one parameter was calculated as (the value of treatment- the value of CDDP)/the value of CDDP × 100%; the expected inhibition (%) was calculated as the observed inhibition of quercetin + the observed inhibition of leucine.

^b^Statistical analysis was performed by the one-sample t-test.

^c^Quercetin alone or in combination with leucine decreased the tumor size while leucine alone increased the tumor size in nude mice exposed to CDDP.
