# Supplementary material for: The combination of quercetin and leucine synergistically improves grip strength by attenuating muscle atrophy by multiple mechanisms in mice exposed to cisplatin
Source: PLoS One. 2023 Sep 12;18(9):e0291462. doi: 10.1371/journal.pone.0291462 (PMC10497166; doi:10.1371/journal.pone.0291462)
Supplement: Attachment — Submitted filename: Reviewe 2.docx [file pone.0291462.s010.docx]

Reviewer #2:

We thank the reviewer for the constructive comments. The following are our point-by-point responses.

1. The combined treatments blunted the decrease in CSA, phosphorylation of Akt and FoxO1, and mTOR signaling of the fast-twitch myofiber-dominant gastrocnemius muscles. Does the combined treatments blunt the decrease in CSA and Akt/mTOR or FoxO1 signaling in the slow-twitch myofiber-dominant soleus muscles?

[ANS]: We did not determine CSA and the expression of Akt/mTOR or FoxO1 signaling in soleus muscles (the slow-twitch myofiber-dominant muscle). The study by Sato et al. (2023) has demonstrated that platinum-based anticancer drugs including cisplatin significantly reduce all the protein levels of MyHCs, including MyHC-I (a slow-twitch myofiber) by the degradation pathway (more details have been described in the answer of the next question). Our study showed that the combined treatments increased the weight of various muscles including soleus muscles compared with the CDDP alone group. Thus, we speculated the combined treatments could regulate those parameters in soleus muscles, too. However, further studies are needed to confirm this speculation and whether they regulated mTOR signaling in the soleus. We have added the information to the Discussion (the last paragraph).

2. Fig 4D: Which types of MyHC were recognized by the anti-MyHC antibodies? Does the combined treatments increase MHC-I, MHC-IIa and MHC-IIb levels?

[ANS]: The MyHC we determined is type IIa. We have added this information to the Methods.

A recent study (Sato et al., 2023) has demonstrated that platinum-based anticancer drugs including cisplatin significantly reduce all the protein levels of MyHC-I, MyHC-IIa and MyHC-IIb, although the anticancer drugs only significantly decrease the gene expression of MyHC-IIa. The authors suggest that this is due to the degradation pathway of muscle proteins being the main mechanism contributing to cisplatin-induced muscle atrophy. Because our study showed that the combined treatments could further decrease the activation of the Akt/FoxO1/MuRF1/atrogin-1 associated protein degradation pathway, we, therefore, speculated that the combined treatments could also increase MyHC-I and MyHC-IIb in muscles. However, more studies are needed to address this possibility. We have now added this information to the Discussion (the 2^nd^ paragraph).

3. Although the gastrocnemius muscles were used in Figs 4, 5, 6, 7, the triceps were used for analyses of glycogen and cytokine levels in Fig 8. In Fig 3, quadriceps were used for analyses of CSA. Please provide a rationale for the used muscle tissues.

[ANS]: Quadriceps, gastrocnemius, and triceps are all fast muscles. Our study showed that CDDP similarly decreased all of these three muscles' weight by about 30% (Table 1). Both quadriceps and gastrocnemius are commonly used to determine CSA, the regulation of proteins associated with protein degradation and synthesis signaling pathways. CDDP has similar effects on those parameters of these two muscle tissues (Sato et al., 2003; Chi et al., 2022). Because the quadriceps muscles were stored in 10% formalin for histological analysis, we used gastrocnemius muscles to conduct western blotting to determine the protein expression of MyHC as well as the molecules which are associated with FoxO1 signaling, mTOR signaling pathway, and E2F-1 signaling pathway. In addition, because the forelimb of the mice was used to measure the MGS, we, therefore, analyzed the glycogen and cytokine levels of the triceps muscle, which is the muscle located in the forelimb. We have now added this rationale to the Discussion (the last paragraph).

4. Table 1: The combined treatments to cisplatin-treated mice completely recovered tissue weight of soleus muscles, but partially tissue weights of triceps, quadriceps, gastrocnemius, and tibialis anterior muscles. Please add the discussion regarding these results.

[ANS]: Based on the suggestion of reviewer #1, we have now used ANOVA with Tukey HSD (instead of Duncan's multiple range) to conduct the statistical analysis for the data in Table 1. The results showed that the combined treatments had better effects on recovering the weight of the quadriceps and soleus (the only slow muscle that we determined) by 29% and 33%, respectively, compared to other muscle tissues. The precise reasons for Q+HL in recovering the soleus muscle remain unclear. The study by Haegens et al. (2012) shows that leucine-induced up-regulation of slow MyHC seems to be better than fast MyHC. The authors found that the mRNA expression of MyHC-7 (the gene encodes MyHC-I) is in an mTOR-independent manner while MyHC-4 (the gene encodes MyHC-IIb) is in an mTOR-dependent manner. Our data also showed that quercetin increased muscle protein synthesis in an mTOR-independent manner. This may explain why the combination of Q and HL had a better recovery effect on the soleus. However, more studies are needed to confirm the mechanisms underlying the effect of Q+HL on the soleus. We have added the information to the Discussion (the last paragraph).

Chi MY, Zhang H, Wang YX, Sun XP, Yang QJ, Guo C. Silibinin Alleviates Muscle Atrophy Caused by Oxidative Stress Induced by Cisplatin through ERK/FoxO and JNK/FoxO Pathways. Oxid Med Cell Longev. 2022; 2022: 5694223.

Haegens A, Schols AM, van Essen AL, van Loon LJ, Langen RC. Leucine induces myofibrillar protein accretion in cultured skeletal muscle through mTOR dependent and -independent control of myosin heavy chain mRNA levels. Mol Nutr Food Res. 2012; 56: 741-752.

Sato K, Miyauchi Y, Xu X, Kon R, Ikarashi N, Chiba Y, Hosoe T, Sakai H. Platinum-based anticancer drugs-induced downregulation of myosin heavy chain isoforms in skeletal muscle of mouse. J Pharmacol Sci. 2023; 152: 167-177.
